# Change of monocytes/macrophages in ulcerative colitis patients with symptoms of anxiety and depression

**DOI:** 10.1186/s12876-023-02693-8

**Published:** 2023-03-11

**Authors:** Xin Gao, Shihao Duan, Yubin Cao, Yan Zhang

**Affiliations:** 1 Department of Gastroenterology, Renmin Hospital of Wuhan University, Wuhan, People’s Republic of China; 2 Department of Gastroenterology, West China Hospital of Sichuan University, No.37 Guoxue Street, Chengdu, 610041 Sichuan People’s Republic of China

**Keywords:** Ulcerative colitis, Monocyte, Macrophage, Macrophage colony-stimulating factor

## Abstract

**Background and aims:**

Monocytes/macrophages play important roles in inflammatory bowel disease and depression, but few studies had focused on the change of monocytes/macrophages in ulcerative colitis (UC) patients with psychiatric disorders.

**Methods:**

UC patients were divided into two groups based on the Hospital Anxiety and Depression Scale (HADS). Demographic and clinical data were captured. Peripheral blood samples and intestinal biopsies were collected for the analysis of monocyte immunophenotype, phagocytic function, and CD4 + T cell differentiation. Transmission electron microscopy was used to observe the ultrastructure of intestinal macrophages.

**Results:**

A total of 139 UC patients were included. 37.41% and 32.37% of UC patients had symptoms of anxiety and depression. In patients with symptoms of anxiety/depression, mayo score, platelet count, erythrocyte sedimentation rate, and endoscopic score, histological scores were significantly higher than those in UC patients without. In patients with symptoms of anxiety/depression, the percentages of CD14 + + CD16 + monocytes and CD14 + CD16++ monocytes were higher, and the phagocytosis was decreased. Patients with symptoms of anxiety/depression had more CD68 + cells and higher M1/M2 ratios in the intestine mucosal layer compared to those without.

**Conclusions:**

Monocytes and intestinal macrophages from UC patients with anxiety/depression tended to polarize to pro-inflammatory subtypes, and their function was also impaired.

**Supplementary Information:**

The online version contains supplementary material available at 10.1186/s12876-023-02693-8.

## Introduction

Depression was an invisible killer. A retrospective analysis showed that anxiety and depression are very common in IBD patients, and those patients are also more likely to need therapy and to utilize healthcare resources [1]. Factors associated with anxiety and depression in IBD patients included disease flares, disabled or unemployed status and socioeconomic deprivation [2], essential psychological interventions would be useful when these factors are identified [3].

Monocytes, which can differentiate into macrophages after migrating to tissues, play important roles in many immune-related diseases [4]. Depressive patients showed a marked alteration in circulating monocytes [5], and macrophages over-activation by social defeat stress can lead to anxiety/depressive-like behaviors [6]. At the same time, the differentiation of monocyte-macrophage was also changed in IBD patients [7]. The number of monocytes and macrophages was increased in the inflamed intestine of IBD patients, and the proportion of proinflammatory monocytes was also increased in active IBD patients [8].

Our previous study showed that peripheral monocytes subpopulation disequilibrium toward intermediate and nonclassical phenotypes, and intestinal macrophage polarization toward M1 phenotype with increased proinflammatory cytokine release were more likely to be found in Crohn’s disease patients with depressive symptoms [9]. However, few studies focused on the change of monocytes/macrophages in UC patients with symptoms of anxiety/depression. This study aimed to analyze the disease characteristics, including life quality, disease activity, and monocyte/macrophage change in UC patients with symptoms of anxiety/depression.

## Methods and materials

### Study design and patient enrollment

Ulcerative colitis patients admitted to West China Hospital, Sichuan University from May 2019 to January 2021 were included in our study. Exclusion criteria were as follows: (1) comorbidities highly associated with anxiety or depression such as carcinoma and cardiovascular disease; (2) concomitant with other chronic or severe psychiatric diseases, like psychosis, psychoactive substance abuse, and dementia; (3) pregnancy; (4) inability to cooperate. In addition, none of our included participants was treated for anxiety or depression. They were divided into two groups, patients with symptoms of anxiety/depression and patients without based on the Hospital Anxiety and Depression Scale (HADS). A HADS score ≥ 8 is indicative of a patient with symptoms of anxiety/depression. Other questionnaires, including the Inflammatory Bowel Disease Questionnaire (IBDQ), the Composite Autonomic Symptom Score (COMPASS)-31, the Fatigue Severity Scale (FSS) and the Pittsburgh Sleep Quality Index (PSQI) were completed at the same time. IBDQ is a disease-specific tool to assess the disease consequences on a patient’s quality of life. The COMPASS-31 is a concise and statistically robust instrument to assess autonomic symptoms that provides clinically relevant scores of autonomic symptom severity. FSS and PSQI were used to evaluate the participants’ fatigue and quality of sleep, respectively. Healthy controls were also involved. Patients’ demographics, clinical disease activity scores (using Mayo score), endoscopic evaluation (using ulcerative colitis endoscopic index of severity, UCEIS) and histological score (Geboes score) were all recorded and compared between the two groups.

### Flowcytometry

Peripheral blood samples were collected on the day of admission. The acquired plasma was stored in aliquots at -80℃ for batched cytokine measurements. Blood samples were processed with modification of a previously described whole-blood technique protocol. After removing erythrocytes, fluorescein isothiocyanate-conjugated anti-CD45 (No. 555482; BD Biosciences), phycoerythrin-conjugated anti-CD16(No. 555407; BD Biosciences), and brilliant violet 510-conjugated anti-CD14(No. 563079; BD Biosciences) were used. The final cell resuspension went through a FACSAria (BD Biosciences) flow cytometer. Monocytes were divided into three subtypes, classical subtype (CD14 + + CD16- cells), intermediate subtype (CD14 + + CD16 + cells) and nonclassical subtype (CD14 + CD16 + + cells). The percentages of monocytes in each subtype were compared. Similar procedures were applied to the measurement of monocyte phagocytosis. Latex beads-rabbit IgG-PE complex was used.

### Coculture of monocyte and CD4 + T cell

CD4 + T cells isolated from peripheral blood mononuclear cells (PBMCs) of healthy volunteers by fluorescence-activated cell sorting (using PE-conjugated anti-CD4, No.555347, on FACSAria, BD Biosciences) were cocultured with monocytes from healthy volunteers, UC patients with symptoms of anxiety/depression and without, respectively. The concentration ratio of CD4 + T cells to monocytes was 5:1. LPS in a final concentration of 100 ng/mL was used in the coculture system. Cells were harvested after 72 h for flowcytometric analysis of Th1/Th2/Th17/Treg proportions.

PE-conjugated anti-CD4, BV711-conjugated anti-CD25 (No.563159, BD Biosciences) and brilliant blue (BB)515-conjugated anti-CD127 (No.564423, BD Biosciences) were used to label Treg cells. PE-cyanine (Cy)7-conjugated anti-IFN-γ, allophycocyanin (APC)-conjugated anti-IL-4 and BV421-conjugated IL-17A (No.557844, No.562438, NO.562933, all from BD Biosciences) with PE-conjugated anti-CD4 were used to detect Th1/Th2/Th17 cells, respectively.

### Luminex assays and enzyme-linked immunosorbent assay (ELISA)

Plasma concentrations of tumor necrosis factor (TNF)-α, IL-6, IL-10, macrophage-colony stimulating factor (M-CSF), granulocyte-macrophage colony-stimulating factor (GM-CSF), and monocyte chemotactic protein-1(MCP-1) were measured by Luminex kit (LXSAHM-10, R&D Systems) according to the manufacturer’s instructions. The level of transforming growth factor (TGF)-β1 was assayed based on the manufacturer’s instruction from Human TGF-β1 ELISA kit (SEKH-0316, G-CLONE).

### Immunofluorescence

Colon biopsy and surgical specimens were achieved and sliced into 4 μm as we described in our previous study. Primary antibodies including anti-CD68 (as a pan-macrophage marker, 1: 100, No. MA5-13324, Invitrogen), anti-CD86 (as a marker for M1 macro-phage, 1: 100, No. MA5-30196, Invitrogen), and anti-CD163 (as a marker for M2 macrophage, 1: 500, No. PA5-78961, Invitrogen) were used.

### Western blot analysis

Proteins were extracted from the colon samples via homogenization in ice-cold lysis buffer. The bicinchoninic acid (BCA) protein assay kit (Thermo) was used to measure the concentrations. Prepared protein samples were separated on 10% sodium dodecyl sulfate polyacrylamide gel electrophoresis (SDS-PAGE) and transferred to polyvinylidene difluoride membranes. Then the membranes were incubated with the anti-M-CSF antibody (1:1000, No. GB11685, Servicebio) overnight at 4 °C. On the following day, membranes were incubated with the secondary antibody (HRP-conjugated Goat anti-Rabbit IgG, No. abs20040, Absin) at room temperature for 2 h. Antibody binding was detected by chemiluminescence using the ECL system (ChemiDoc MP, Bio-Rad). Densitometry of the blots was analyzed with Image Lab 5.2 software.

### Reverse transcription-polymerase chain reaction

For qRT analyses, colon samples were lysed immediately after using the TRIzol Reagent (Life Technologies), and total RNA isolation was performed according to the manufacturer’s protocol. cDNA was generated using EuroScript Reverse Transcriptase (Euroclone Cytogenetics), with random examers and 2.5 µg RNA per reaction. qRT-PCR reactions were then prepared with the PowerUp SYBR Green Mix (Applied Biosystems) and run using a Bio-Rad CFX Maestro system (Applied Biosystems). Primer pairs were designed *ex novo* with NCBI Primer-BLAST. Verification and location of target gene sequences were performed on Ensembl Genome Browser. Primer sequences of M-CSF are listed as follows: (5'-3') F: CCTGCTGTTGTTGGTCTGTCTC, R: GGTACGAGGTCTCCATCTGA. Results were normalized using GAPDH as housekeeping gene as a reference and evaluated using the 2^−ΔΔCt^ method.

### Statistical analysis

All data were analyzed with SPSS 22.0 and GraphPad Prism 6.0 software. The Kolmogorov–Smirnov test was performed to demonstrate if the data were in normal distribution. Continuous variables were presented as median values (interquartile range [IQR]), while categorical variables were presented as percentages. Comparisons between the 2 groups were made using the Mann–Whitney U test for continuous variables or with the Pearson chi-square test for categorical variables. Comparisons among multiple groups were done with the Kruskal–Wallis test as well as Dunn’s multiple comparisons test. Correlations between 2 variables were assessed with Spearman’s rank correlation coefficient. Statistical significance was considered achieved for P < 0.05.

## Results

### Ulcerative colitis patients with symptoms of anxiety/depression had worse quality of life

139 UC patients were included and were evaluated based on HADS. 37.41% (51/139) of UC patients had the symptoms of anxiety, and 32.37% (45/139) had the symptoms of depression. UC patients with either one of two symptoms, anxiety or depression, or with both were defined as UC patients with symptoms of anxiety/depression, including 15 cases only with symptoms of anxiety, 8 cases only with symptoms of depression and 37 cases with both. In total, 43.17% (60/139) of UC patients had symptoms of anxiety/depression.

Patients were divided into two groups, UC patients with symptoms of anxiety/depression and patients without. UC patients with symptoms of anxiety/depression were older than UC patients without [48(33,55) vs. 32(24,51), P = 0.0012], and had lower education level (primary school and junior school graduation or even below,43.33% vs. 26.58%, P = 0.0164). There were no significant differences in the gender ratio, BMI or disease duration between the two groups (P = 0.5114, 0.7696 and 0.0969, respectively) (Table 1).

**Table 1:** Demographic characteristics of the study population

Variable	UC patients without symptoms of anxiety/depression (n = 79)	UC patients with symptoms of anxiety/depression (n = 60)	P-value
Age (year), median (IQR)	32(24,51)	48(33,55)	0.0012*
Male, n (%)	43(54.43%)	36(60.00%)	0.5114
*Education status*			
Primary and below	2(2.53%)	9(15.00%)	0.0164*
Middle school	19(24.05%)	17(28.33%)	
High school	27(34.18%)	21(35.00%)	
Bachelor or above	31(39.24%)	13(21.67%)	
BMI (kg/m^2^), median (IQR)	20.30(18.25,22.00)	20.67(18.27,22.04)	0.7696
Duration of disease(month), median (IQR)	24(8,60)	36(16,97)	0.0969
*Smoking use, n(%)*			
Current	0(0.00%)	4(6.67%)	0.0173*
Quitted	13(16.46%)	14(23.33%)	
Never	66(83.54%)	42(70.00%)	
Surgery, n(%)	3(3.80%)	8(13.33%)	0.0809
*Montreal classfication, n (%)*			
E1	6(7.59%)	1(1.67%)	0.2454
E2	23(29.11%)	16(26.67%)	
E3	50(63.29%)	43(71.67%)	
*Extraintestinal manifestations, n (%)*	10(12.66%)	16(26.67%)	0.0359*
Mayo score, median (IQR)	8(6,9)	10(8,11)	< 0.0001*
*Laboratory examination*			
White blood cell (10^9^/L), median (IQR)	7.24(5.24,8.83)	7.39(5.91,9.65)	0.3234
Neutrophil count(10^9^/L), median (IQR)	4.97(3.20,6.06)	4.61(3.49,6.82)	0.5851
Neutrophil ratio (%), mean ± SD	65.77 ± 13.27	65.67 ± 12.88	0.9656
Lymphocyte count (10^9^/L), median (IQR)	1.40(1.08,2.02)	1.71(1.20,2.01)	0.2061
Lymphocyte ratio (%), mean ± SD	23.97 ± 11.32	24.04 ± 10.43	0.7740
Monocyte count(10^9^/L), median (IQR)	0.43(0.33,0.58)	0.49(0.38,0.72)	0.0752
Monocyte ratio (%), median (IQR)	6.5(5.1,8.9)	7.0(5.6,9.3)	0.2629
Hemoglobin (g/L), median (IQR)	109(86,131)	108(90,127)	0.9282
Platelet (10^9^/L), median (IQR)	256(202,310)	301(221,398)	0.0222*
C-reactive protein(mg/L), median (IQR)	11.1(2.9,34.3)	17.5(4.5,56.2)	0.1594
ESR (mm/h), median (IQR)	27(16,50)	41(26,69)	0.0062*
Albumin(g/L), mean ± SD	38.27 ± 7.11	34.79 ± 7.51	0.0061*
Creatinine(μmol/L), median (IQR)	61(50,72)	65(55,78)	0.1554
ASCA positive, n(%)	39(49.37%)	27(45.00%)	0.6096
UCEIS, mean ± SD	4.620 ± 1.727	6.033 ± 1.775	< 0.0001*
Geboes, median (IQR)	4.1(3.1,4.2)	5.1(4.2,5.2)	< 0.0001*
*Current treatment*^*a*^			
5-Aminosalicylic, n(%)	58(73.42%)	40(66.67%)	0.3873
Corticosteroids, n(%)	23(29.11%)	35(58.33%)	0.0005*
Thiopurines, n(%)	11(13.92%)	14(28.33%)	0.9259
Anti-TNF, n(%)	9(11.39%)	5(8.33%)	0.5528

*IQR* interquartile range; *BMI* body mass index; Montreal classification: E1 refers to the involvement limited to the rectum; E2 refers to the involvement limited to the proportion of the colon distal to the splenic flexure; E3 refers to the involvement extends proximal to the splenic flexure, including pan-colitis; *ESR* erythrocyte sedimentation rate; *ASCA* anti-saccharomyces cerevisiae antibody; *UCEIS* ulcerative colitis endoscpic index of severity

^*a*^treatment used during hospitalisation at enrollment

*P < 0.05

**Fig. 1:**
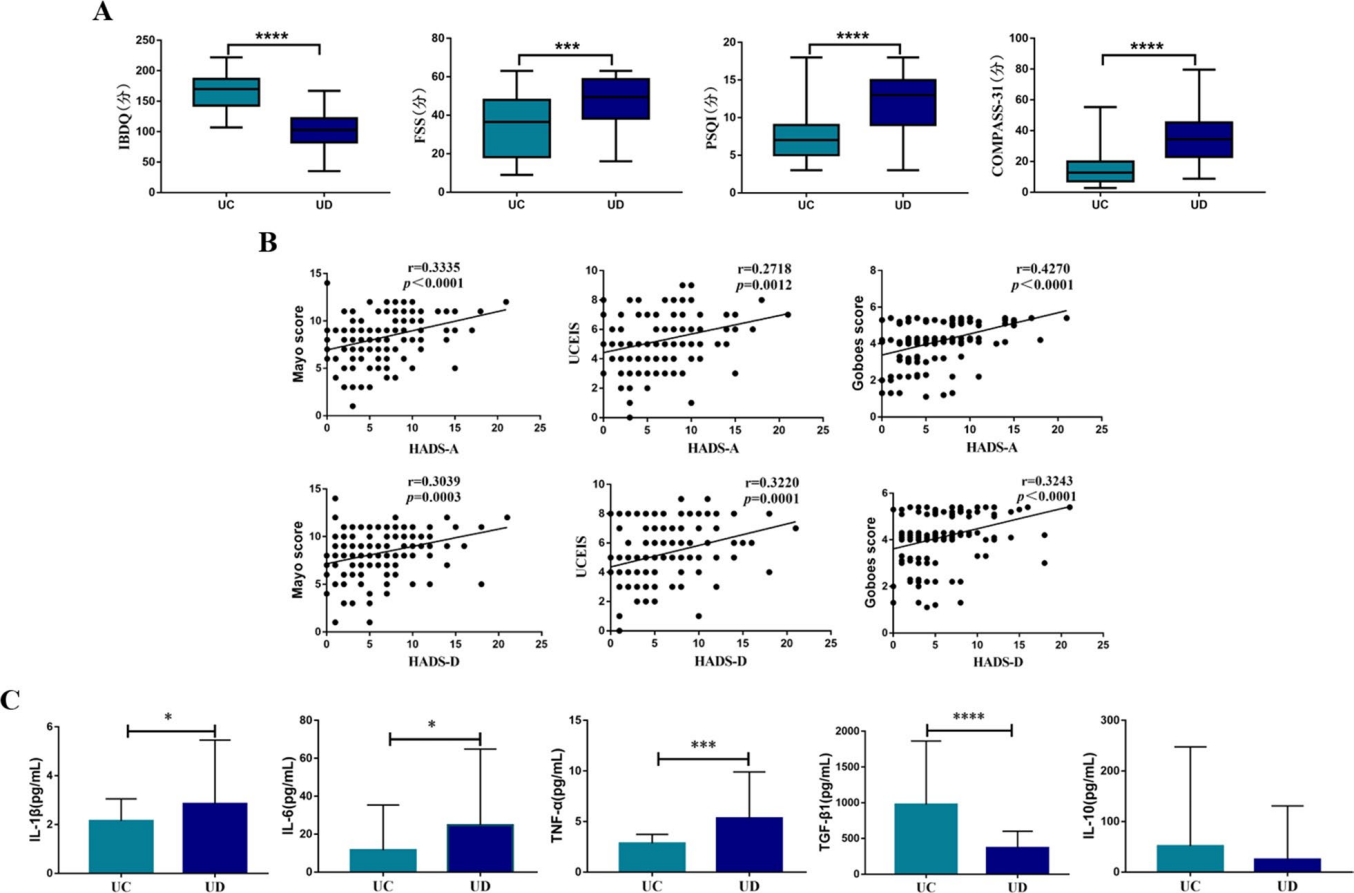
Impact of symptoms of anxiety/depression on UC patients. **A** Comparison of IBDQ scores, FSS scores, PSQI scores and Compass-31 scores between UC (n = 32) and UD (n = 28) patients; **B** the relationship between the disease activity (Mayo score, UCEIS, Goboes score) of the two groups and the scores of hospital anxiety and depression scale. **C** Comparison of proinflammatory cytokines, IL-1β, IL-6 and TNF-α, and anti-inflammatory cytokines, TGF-β1, IL-10, between UC (n = 22) and UD (n = 28) patients. UC: UC patients without symptoms of anxiety/depression; UD: UC patients with symptoms of anxiety/depression; IBDQ: Inflammatory bowel disease questionnaire; FSS: Fatigue severity Scale; PSQI: Pittsburgh Sleep Quality Index; Compass-31: Composite Autonomic symptom score-31; *P < 0.05, ***P < 0.001, ****P < 0.0001

IBDQ score was lower, and scores of FSS, PSQI and COMPASS-31 were higher in UC patients with symptoms of anxiety/depression compared to UC patients without symptoms of anxiety/depression, indicating the worse quality of life (P < 0.05, Fig. 1A).

### Ulcerative colitis patients with symptoms of anxiety/depression suffered more severe disease

In patients with symptoms of anxiety/depression, mayo score, platelet count, erythrocyte sedimentation rate, and endoscopic score were significantly higher than those in UC patients without symptoms of anxiety/depression (P < 0.05), and UC patients with symptoms of anxiety/depression were prescribed steroids more frequently (58.33% vs. 29.11%, P = 0.0005). Histological score (Geboes score) was also higher in UC patients with symptoms of anxiety/depression [5.1(4.2,5.2) vs. 4.1(3.1,4.2), P < 0.0001] (Table 1).

Moreover, we further analyzed the correlation between the disease activity of the two groups and the scores of hospital anxiety and depression scale. Results showed that mayo score, UCEIS score and Geboes score were positively correlated with anxiety symptom scores in HADS (P < 0.0001, 0.0012, < 0.0001, respectively). Similarly, mayo score, UCEIS score and Geboes score were positively correlated with depressive symptom scores in HADS (P = 0.0003, 0.0001, < 0.0001, respectively) (Fig. 1B).

The plasma levels of proinflammatory cytokines, including IL-1β、IL-6 and TNF-α were significantly higher in UC patients with symptoms of anxiety/depression, while the level of anti-inflammatory cytokine TGF-β1 was evidently lower in UC patients with symptoms of anxiety/depression compared to UC patients without symptoms of anxiety/depression (P < 0.05, Fig. 1C).

### Monocytes in ulcerative colitis patients with symptoms of anxiety/depression tended to differentiate into pro-inflammatory phenotypes

**Fig. 2:**
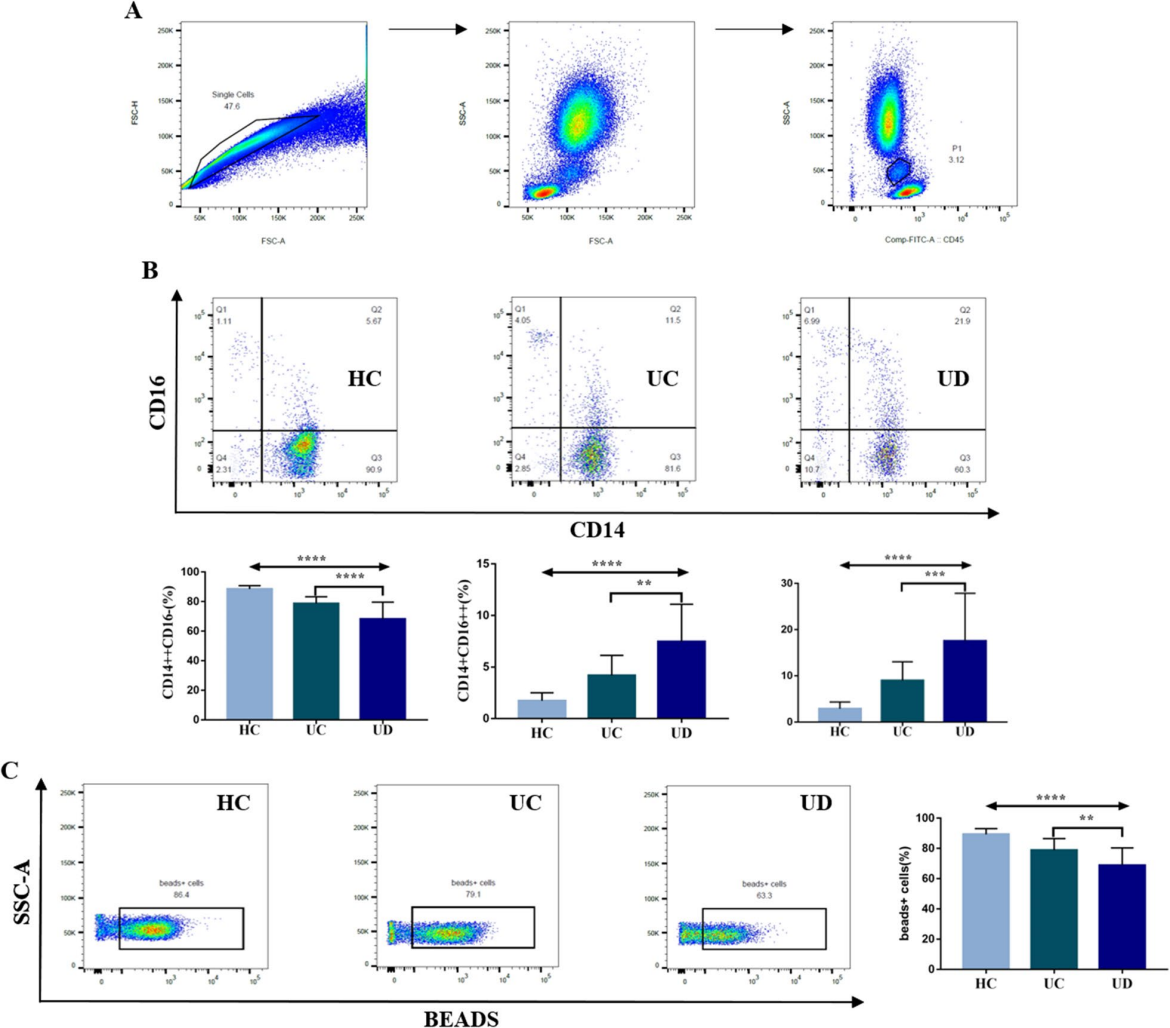
Immunophenotypes and phagocytosis of monocytes analyzed by flow cytometry. **A** FSC-A/FSC-H showed the circle effective single cells, FSC-A/SSC-A showed the population of peripheral blood mononuclear cells (PBMC), CD45/SSC showed the expression of CD45, cells in P1 were monocytes; **B** the proportions of classic, intermediate and nonclassical monocytes in the HC, UC and UD groups were compared, respectively. CD14 and CD16 are used as markers to distinguish between classical, intermediate and non-classical monocyte immunophenotypes. **C** Beads + cells represented monocytes that engulfed beads in HC, UC, and UD, respectively. Monocyte phagocytosis was compared among the three groups; HC: healthy controls, n = 13; UC: UC patients without symptoms of anxiety/depression, n = 19; UD: UC patients with symptoms of anxiety/depression, n = 23, **P < 0.01, ***P < 0.001, ****P < 0.0001

The gating strategies for monocytes in peripheral blood was shown in Fig. 2A. The percentages of intermediate monocytes (CD14 +  + CD16 + monocytes) and nonclassical monocytes (CD14 + CD16 +  + monocytes) were higher in UC patients with symptoms of anxiety/depression [17.29(10.00,21.40) vs. 8.73(6.26,11.50), P = 0.0004; 7.46 ± 0.76 vs. 4.18 ± 0.45, P = 0.0010] (Fig. 2B). Phagocytosis was significantly impaired in UC patients with symptoms of anxiety/depression compared to UC patients without (68.88 ± 2.39 vs. 78.79 ± 1.78, P = 0.0027) (Fig. 2C).

**Fig. 3:**
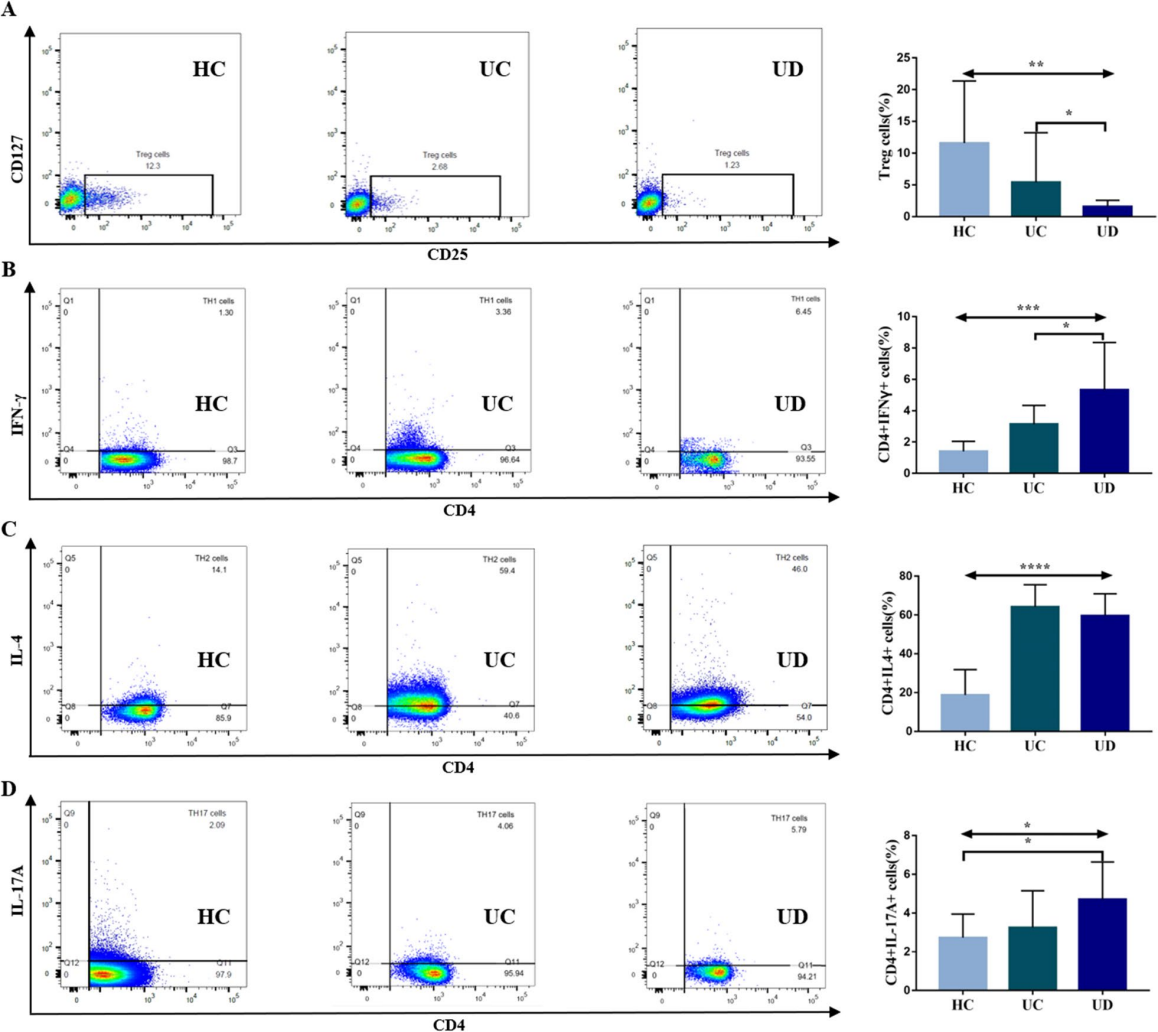
Effect of monocytes from UC patients on the polarization of CD4 + cells towards TH1/2/17 cells. **A** The proportions of Treg cells in CD4 + T cells in HC, UC and UD patients, CD4 + CD25 + CD127- in the box was defined as Treg cells in the CD4 + T cell population; **B** the proportions of Th1 cells in CD4 + T cells in HC, UC and UD patients, CD4 + IFN-γ + in the box was defined as Th1 cells in the CD4 + T cell population; **C** the proportions of Th2 cells in CD4 + T cells in HC, UC and UD patients, CD4 + IL-4 + in the box was defined as Th2 cells in the CD4 + T cell population; **D** the proportions of Th17 cells in CD4 + T cells in HC, UC and UD patients, CD4 + IL-17A + in the box was defined as Th17 cells in the CD4 + T cell population. HC: healthy controls, n = 9; UC: UC patients without symptoms of anxiety/depression, n = 9; UD: UC patients with symptoms of anxiety/depression, n = 12. *P < 0.05, **P < 0.01

Monocytes from UC patients with symptoms of anxiety/depression significantly inhibited CD4 + T cells from healthy volunteers polarized to Treg cells [1.37(0.68,2.34) vs. 2.40(2.15,4.56), P = 0.0124] (Fig. 3A), and induced CD4 + T cells to differentiate into Th1 cells [4.52(3.05,6.37) vs. 3.25(2.07,3.77), P = 0.0409] (Fig. 3B) contrasted to monocytes from UC patients without symptoms of anxiety/depression. However, the effects of monocytes on the polarization of CD4 + T cells to TH2 cells (Fig. 3C) and TH17 cells (Fig. 3D) were comparable between UC patients with and without symptoms of anxiety and depression.

### The change of intestinal macrophage in ulcerative colitis patients with symptoms of anxiety/depression

**Fig. 4:**
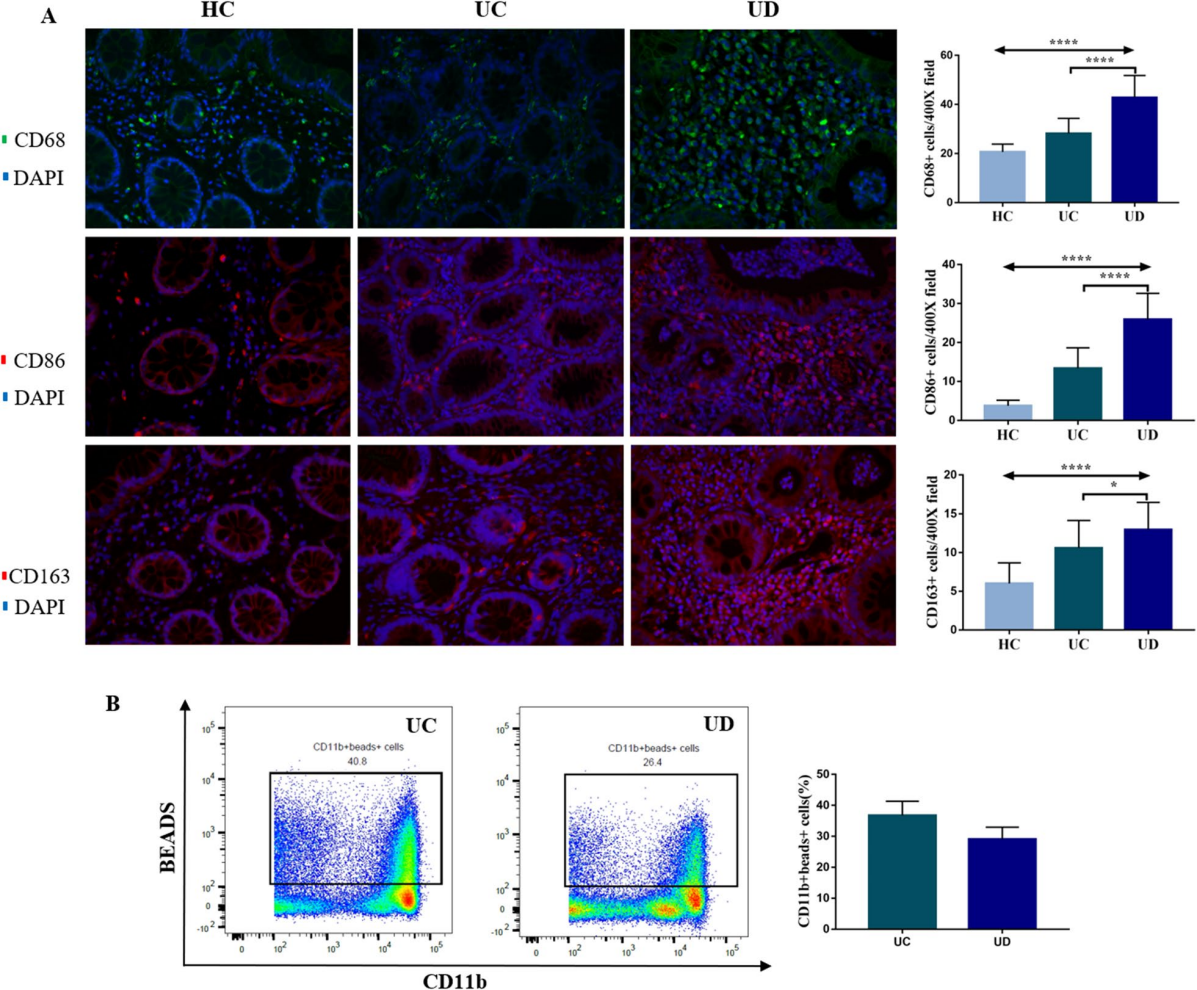
Immunophenotypes and phagocytosis of intestinal macrophages analyzed by immunofluorescence and flow cytometry. **A** The total number of macrophages (CD68 + cells), M1 macrophages (CD86 + cells), and M2 macrophages (CD163 + cells) in the intestinal mucosa of HC (n = 5), UC (n = 10) and UD (n = 10) patients; **B** phagocytosis of intestinal lamina propria macrophages in UC (n = 3) and UD (n = 2) patients. HC: healthy controls; UC: UC patients without symptoms of anxiety/depression; UD: UC patients with symptoms of anxiety/depression. Figures were taken under 400 × magnification field of vision. *P < 0.05, ****P < 0.0001

The total number of intestinal mucosal macrophages was increased in UC patients compared to that in healthy controls (P < 0.05). UC patients with symptoms of anxiety/depression had a larger number of CD68 + cells in the intestine mucosal layer compared to UC patients without symptoms of anxiety/depression [43(38,49) vs. 29(26,31), P < 0.0001]. And the ratio of M1/M2 was higher in UC patients with symptoms of anxiety/depression (2.15 ± 0.18 vs. 1.31 ± 0.11, P = 0.0004) (Fig. 4A). Phagocytosis of intestinal lamina propria macrophages in UC patients with symptoms of anxiety/depression was impaired, but the difference showed no statistical significance (29.10 ± 2.70 vs. 36.71 ± 2.63, P = 0.1489) (Fig. 4B).

### M-CSF may play a role in monocyte/macrophage change in ulcerative colitis patients with symptoms of anxiety/depression

**Fig. 5:**
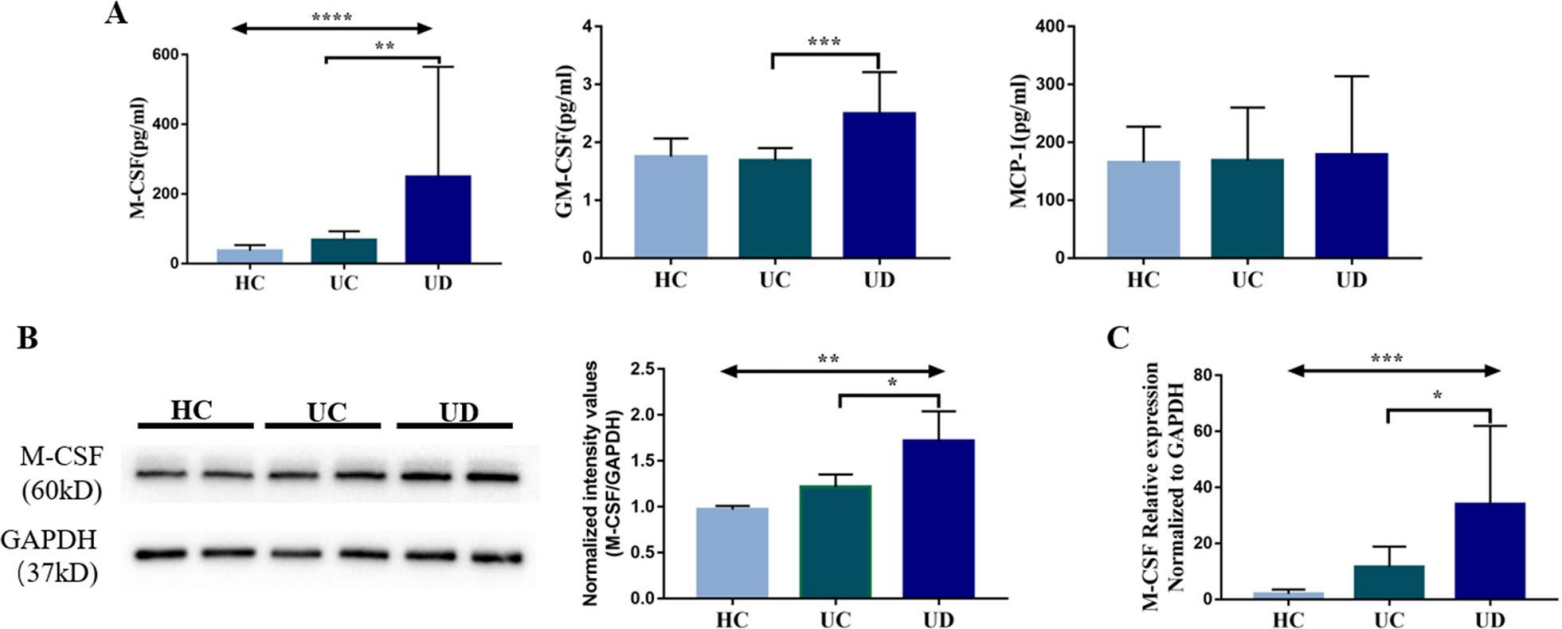
Elevated plasma and intestinal M-CSF levels in UC patients with symptoms of anxiety/depression. **A** The plasma M-CSF, GM-CSF and MCP-1 levels detected by Luminex in HC (n = 10), UC (n = 16) and UD (n = 20) patients; **B** the expression of intestinal M-CSF protein in HC (n = 4), UC (n = 4), UD (n = 4) patients. The GAPDH and M-CSF protein expression was detected by Western-Blots. Full-length blots/gels are presented in Additional file 1: Fig. S1; **C** the relative expression levels of M-CSF RNA in HC (n = 4), UC (n = 5) and UD (n = 6) patients. HC: healthy controls; UC: UC patients without symptoms of anxiety/depression; UD: UC patients with symptoms of anxiety/depression. *P < 0.05, **P < 0.01, ***P < 0.001, ****P < 0.0001

The renewal of intestinal macrophages depends on the continuously supplemented of peripheral blood monocytes. We compared the cytokines involved in monocyte/macrophage differentiation among groups, including M-CSF, GM-CSF and MCP-1. The levels of M-CSF and GM-CSF in plasma of patients with ulcerative colitis with symptoms of anxiety/depression were significantly higher than those in patients with ulcerative colitis without symptoms of anxiety/depression [M-CSF:118.20(64.85, 154.10) vs. 65.16(47.28, 83.81), P = 0.0057; GM-CSF: 2.49 ± 0.16 vs. 1.69 ± 0.05, P = 0.0001], while there was no significantly difference in MCP-1 level (pg/mL) between the two groups [139.3(98.4, 176.9) vs. 144.5(107.9, 192.8), P = 0.7983] (Fig. 5A). Further, the protein level of intestinal M-CSF was analyzed and showed that the level in patients with ulcerative colitis accompanied by anxiety/depression was significantly higher than that in patients without anxiety/depression (1.72 ± 0.16 vs. 1.22 ± 0.07, P = 0.0295) (Fig. 5B). The expression of M-CSF at the RNA level was also significantly increased (33.88 ± 28.11 vs. 11.55 ± 7.29, P = 0.0303) (Fig. 5C).

## Discussion

Studies have verified that IBD is closely related to mental disorders, including anxiety and depression [1, 10, 11]**.** Experts even called for examining the psychological states while treating IBD [3]. Data from the National Healthcare Insurance service in Korea showed that the cumulative incidences of anxiety and depression were elevated with a steep rise after the diagnosis of UC [12]. A systematic review concluded that the rate of anxiety in UC patients was 31%, depression was 22% [13], while a Swiss IBD study indicated that the rate of anxiety or depression in UC patients were 34.9%, 18.5% respectively [14]. The incidence of symptoms of anxiety/depression in UC patients in our study was consistent with the above studies.

In our study, UC patients with symptoms of anxiety/depression suffered more severe diseases, including worse quality of life, higher scores of mayo score and Geboes score. The concentrations of proinflammatory cytokines, like TNF-α, IL-1β and IL-6 were significantly higher in UC patients with symptoms of anxiety/depression. These results suggested that UC patients with symptoms of depression/anxiety had higher levels of proinflammatory cytokines. Another study showed that depressed patients had increased levels of proinflammatory cytokines, and inflammatory responses participated in the pathophysiology of depression [15]. Therefore, we speculate that depression may aggravate UC through releasing proinflammatory cytokines, but the relationship between depression and UC, and the roles of proinflammatory cytokines need further research.

Monocytes play an important role in innate inflammatory responses with different immunophenotypes in immunoreaction. Depressive patients had a significant increase proportion of intermediate monocytes and a decreased proportion of classical monocytes compared to healthy controls [5]. Another study showed that UC patients had more intermediate and non-classical monocytes, and fewer classical monocyte [16]. Those results were consistent with our conclusions. Monocyte may act as scavenger cells to phagocytic dead cells, debris and recognize pathogens. Researchers explored phagocytosis among three immunophenotypes and found that classical monocyte exhibited significantly higher phagocytic capacity compared to intermediate and non-classical monocytes [17]. We found that UC patients with symptoms of anxiety/depression had impaired phagocytosis, which may be related to the change of immunophenotype. Reduced phagocytic capacity was not only unable to effectively eliminate pathogens, but also limit its antigen presentation ability [18]. Our study also showed that monocytes from UC patients with symptoms of anxiety/depression inhibited CD4 + T cells polarized to Treg cells, but induced CD4 + T cells to differentiate into Th1 cells, modulating the immune response. Those may explain why UC patients with symptoms of anxiety/depression had a worse course.

Intestinal macrophages are key players in IBD. The lamina propria of the inflamed intestine in patients with IBD was massively infiltrated by CD68 cells M1 macrophages [18]. Lamina propria monocytes and M1 macrophages invaded intestinal tissues, resulting in epithelial barrier impairment and driving intestinal inflammation in IBD [8]. Our study found that UC patients with symptoms of anxiety/depression had more macrophages in the intestine, especially M1 macrophages, but the mechanism of how monocytes transform into macrophages remains unclear.

Unlike liver Kupffer cells, or brain microglial cells, intestinal macrophages require peripheral blood monocyte constantly replenish, but not self-proliferation in situ [19]. So, we compared key factors in the differentiation of monocytes into macrophages, including M-CSF, GM-CSF and MCP-1. In active IBD, the number of M-CSF-expressing cells was significantly increased and their distribution markedly altered [20]. M-CSF-deficient osteopetrotic mice (op/op) appeared less vulnerable to colitis induced by DSS. Macroscopic damage, microscopic injury, MPO activity, and tissue concentrations of TNF-alpha, IL-1beta, and IL-6 were all lower in op/op mice compared with M-CSF-expressing heterozygote (+/?) mice with DSS colitis, indicating that M-CSF-dependent macrophages played a pro-inflammatory role in colitis [21]. Our study found that serum and intestinal levels of M-CSF in UC patients with symptoms of anxiety/depression were increased, which may suggest that M-CSF may be related to the change in monocyte/macrophage differentiation and function in the UC patients with symptoms of anxiety/depression, but more details are needed.

## Conclusions

In a word, our study found that UC patients with symptoms of anxiety/depression suffered worse disease, which may be related to the change of phenotypes and functions of monocyte/macrophage. And M-CSF may participate in the latter, but further study needs to be carried out.

## Supplementary Information

**Additional file 1. Figure S1.** Full-length blots/gels of intestinal M-CSF and GAPDH protein in HC, UC and UD patients.

## Data Availability

All data generated or analysed during this study are included in this published article (and its Additional file 1).
