# Supplementary material for: Change of monocytes/macrophages in ulcerative colitis patients with symptoms of anxiety and depression
Source: BMC Gastroenterol. 2023 Mar 11;23:67. doi: 10.1186/s12876-023-02693-8 (PMC10007821; doi:10.1186/s12876-023-02693-8)
Supplement: Supplementary file 1 — Additional file 1. Figure S1. Full-length blots/gels of intestinal M-CSF and GAPDH protein in HC, UC and UD patients. [file 12876_2023_2693_MOESM1_ESM.pptx]

## Slide 1
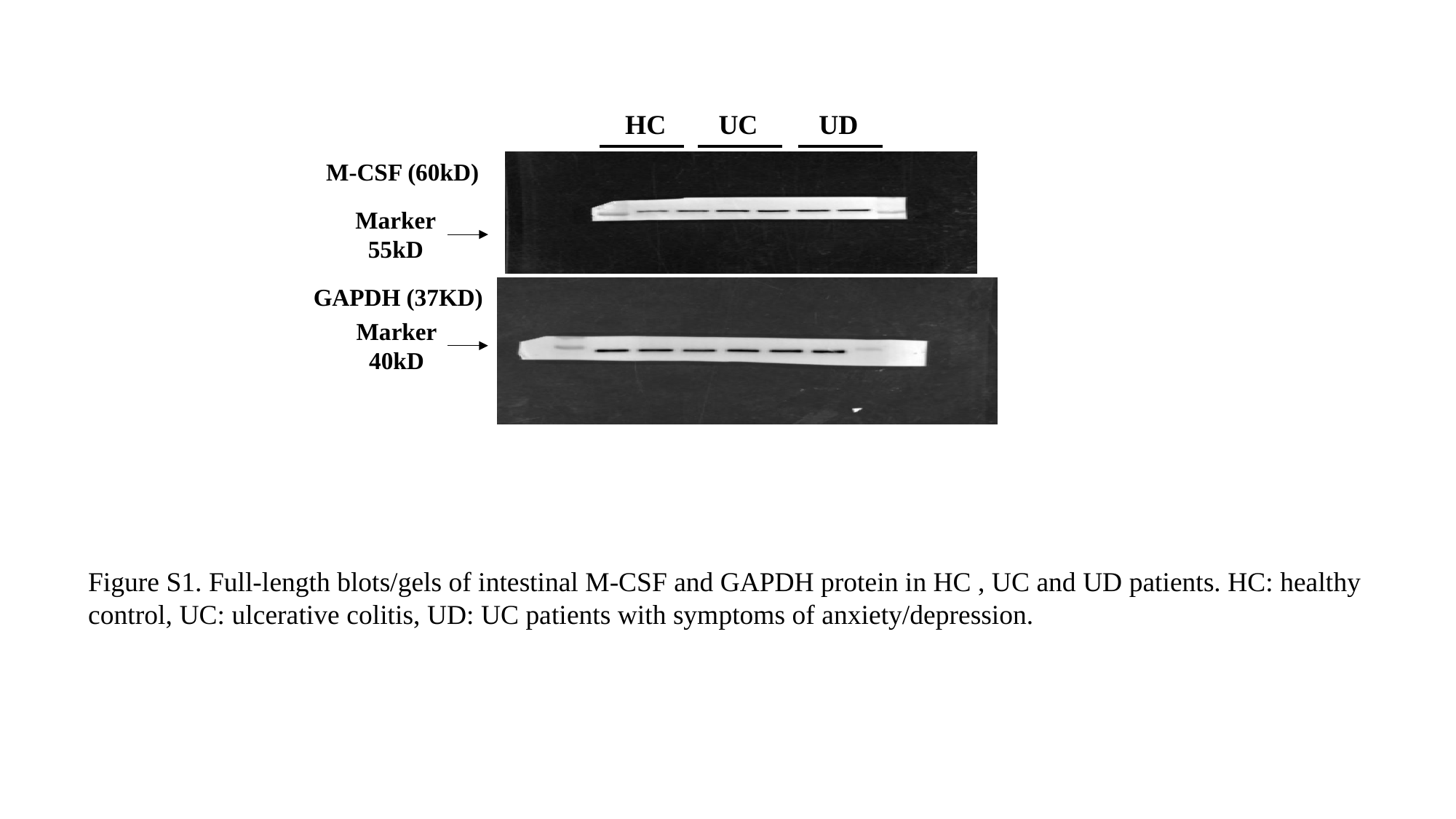

HC
UC
UD
M-CSF (60kD)
Marker
55kD
GAPDH (37KD)
Marker
40kD
Figure S1. Full-length blots/gels of intestinal M-CSF and GAPDH protein in HC , UC and UD patients. HC: healthy control, UC: ulcerative colitis, UD: UC patients with symptoms of anxiety/depression.
